# Stressor-Like Effects of c-Jun N-Terminal Kinase (JNK) Inhibition

**DOI:** 10.1371/journal.pone.0044073

**Published:** 2012-08-29

**Authors:** Melanie Clarke, Rowan Pentz, Jessica Bobyn, Shawn Hayley

**Affiliations:** Department of Neuroscience, Carleton University, Ottawa, Ontario, Canada; Chiba University Center for Forensic Mental Health, Japan

## Abstract

There is an urgent need for novel treatment strategies for stressor related disorders, particularly depression and anxiety disorders. Indeed, existing drug treatments are only clinically successful in a subset of patients and relapse is common. This likely stems from the fact that stressor disorders are heterogeneous with multiple biological pathways being affected. To this end, the present investigation sought to assess in mice the contribution of the c-Jun N terminal kinase (JNK) pathway to the behavioral, hormonal and neurochemical effects of an acute stressor. Indeed, although JNK has been shown to modulate glucocorticoid receptors *in vitro*, virtually nothing is known of the role for JNK in affecting stressor induced pathology. We presently found that the JNK antagonist, SP600125, (but not the p38 antagonist, SB203580) increased plasma corticosterone levels under resting conditions and in the context of an acute stressor (wet bedding + restraint). SP600125 also reduced exploration in an open field arena, but prevented the stressor induced increase in open arm exploration in an elevated plus maze. Finally, SP600125 affected noradrenergic activity in the central amygdala and locus coruleus under resting condition, but prevented the noradrenergic effects within the paraventricular nucleus of the hypothalamus that were induced by the acute stressor exposure. These data suggest inhibiting endogenous JNK can have stressor-like corticoid, behavioral and central monoamine effects under basal conditions, but can actually reverse some behavioral and neurochemical effects of an acute stressor. Thus, endogenous JNK appears to affect stress relevant processes in a context-dependent manner.

## Introduction

Stressor exposure is the primary environmental challenge believed to contribute to a range of neuropsychiatric conditions, most notably clinical depression and anxiety disorders [1], [2], [3]. Indeed, repeated or particularly severe exposure to psychogenic or neurogenic stressors induces enduring pathological hormonal and neurotransmitter changes that culminate in behavioral pathology [4], [5]. That said, even more modest stressor exposure can induce profound activation of the hypothalamic pituitary-adrenal (HPA) axis, coupled with augmented monoamine turnover in a variety of limbic and cortical brain regions [6], [7], [8], [9]. Over time, such stressor induced changes can lead to allostatic overload and render individuals vulnerable to subsequent stressful situations [10]. However, very little is currently known regarding the underlying intracellular pathways that mediate stressor induced hormonal and neurochemical alterations.

Given the marked peripheral and central effects of stressor induced glucocorticoids, it is of tremendous clinical importance to better understand the pathways regulating their receptors and their downstream consequences. In this regard, functioning of the glucocorticoid receptor (GR) is normally modulated by phosphorylation at serine and threonine residues by a variety of enzymes, including mitogen-activated protein (MAP) kinases [11]. Of the MAP kinases, c-Jun N-terminal kinase (JNK; also known as stress activated protein kinases) might be particularly relevant for stressor situations given the recent *in vitro* findings showing JNK to physically interact with the glucocorticoid receptor (GR) and negatively regulate its expression and transcriptional activity [12], [13]. The JNKs are encoded by three genes, of which JNKs 1 and 2 are ubiquitously expressed, whereas JNK 3 expression occurs primarily in the brain [14]. JNK signaling is known to be involved in a wide range of physiological functions, including inflammatory and proliferative responses induced by infection, pro-inflammatory cytokines, and cellular stress [15], [16].

Although scant data are available concerning the role of JNK in the context of psychogenic or neurogenic stressors, one recent study did report that JNK phosphorylated GRs in response to restraint stress [17]. Accordingly, treatment of mouse hippocampal cells with a selective JNK inhibitor, SP600125, enhanced GR activity [13]. These findings are consistent with JNK normally having a tonic inhibitory role on GR function. To this end, we currently hypothesized that pharmacological inhibition of JNK would modify the behavioral, corticosterone and central monoamine alterations induced by an acute stressor (wet bedding + restraint) treatment. In fact, we found that the JNK inhibitor, SP600125, itself actually induced stressor-like effects, such that plasma corticosterone levels and noradrenergic activity within the central amygdala and locus coruleus were increased and open-field performance altered. However, SP600125 reversed the hypothalamic noradrenergic and elevated plus maze changes induced by the acute stressor. These data support the contention that endogenous JNK might normally have tonic inhibitory effects upon HPA, central monoamine and behavioral activity. However, in the context of an acute stressor, JNK might play an excitatory role for certain behaviors and neurotransmitter systems.

## Methods

### 2.1 Animals

Male CD1 mice were purchased from Charles River Laboratories (Laprairie, Quebec, Canada) at 8–10 weeks of age. Mice were singly housed in standard polypropylene cages (27×21×14 cm), and maintained on a 12-hour light/dark cycle (light phase: 0700–1900 h). Water and Ralston Purina mouse chow (St. Louis, MO, USA) were provided *ad libitum*, and room temperature was maintained at 21°C. Animals were acclimatized to the laboratory for a period of one week before experimental procedures were commenced. All experiments were approved by the Carleton University Committee for Animal Care and adhered to the guidelines outlined by the Canadian Council for the Use and Care of Animals in Research. Mice were divided into four treatment groups as follows: 1.vehicle + stress, 2. vehicle + no stress, 3. JNK antagonist + no stress, 4. JNK antagonist + stress. Half of the mice from each group underwent rapid decapitation and their trunk blood and brains were collected for monoamine analysis. The other half was used for behavioral assessment.

A separate cohort of 48 mice was used to determine if any effect of JNK inhibition on corticosterone levels was specific to this kinase, or whether the alternate MAP kinase, p38, might also have such consequences. Indeed, p38 has recently been implicated in depressive-like behavioral and neurochemical pathology [18]. To this end, the treatment groups were as follows: 1.vehicle + stress, 2. vehicle + no stress, 3. JNK antagonist + no stress, 4. JNK antagonist + stress, 5. p38 antagonist + no stress, 6. p38 antagonist + stress.

### 2.2 Treatments

Mice were given an intraperitoneal (i.p.) injection of either SP600125 (30 mg/kg; JNK antagonist), SB203580 (1 mg/kg; p38 antagonist) or vehicle (DMSO diluted with saline to a ratio of 1∶4). Fifteen minutes later the mice in the stress group had their bedding wetted with 250 ml of water. After 10 minutes of wet bedding, these mice were immediately placed into a “restraint bag” for 15 minutes. The restraint apparatus consisted of a conical shaped plastic bag with an opening at the end to allow breathing. The animals were placed snugly in the plastic bag with their tails taped down to prevent excessive movement. Mice therefore had a total stress exposure of 25 minutes. Five minutes after being removed from the restraint bag, the mice were either rapidly decapitated and their trunk blood and brains kept for analysis, or subjected to behavioral testing. End points were therefore 45 minutes from the time of vehicle or drug injection.

### 2.3 Behavior

Five minutes after the termination of the stressor exposure, mice were placed into a clear, Plexiglas open-field arena (40×40 cm), which was illuminated by ambient fluorescent ceiling lights. Mice were placed in one corner of the arena and allowed to freely explore the space for 10 minutes. Activity was recorded by a video camera that was mounted directly above the open-field apparatus. The video camera was connected to tracking software (EthoVision, Noldus, Netherlands) which scored latency to enter the center, time spent and number of entries into the center square, middle region, and outer square.

Immediately after the open-field exposure, mice were subjected to a plus-maze test for 5 minutes in duration. Specifically, mice were placed into the center of the Plexiglas apparatus (30 cm high) which was composed of four arms (6 cm×30 cm), two of which were enclosed by walls and two of which were open. Time spent in and number of entries into each of the four arms was recorded by an experimenter blind to the animal groups. To be scored as an entry into an arm, all four paws had to be placed into that arm.

### 2.4 Trunk Blood and Brain Tissue Collection

Forty-five minutes after the injection, mice were rapidly decapitated and trunk blood and brains were collected. Brain regions were micro-punched from coronal brain sections that were obtained using a chilled microdissecting block that contained slots (0.5 mm apart) for single edged razor blades. The prefrontal cortex (PFC), central amygdala (CeA), hippocampus (HC), paraventricular nucleus (PVN) and locus coeruleus (LC) were removed using microdissection needles. The tissue was immediately placed in a homogenizing buffer containing 14.17 g monochloroacetic acid, 0.0186 g EDTA, 5.0 mL methanol and 500 mL H_2_O, and was stored at −80°C until high-performance liquid chromatography (HPLC). For Western blot analysis, tissue was collected in empty tubes and immediately stored at −80°C until analysis was performed.

### 2.5 Corticosterone Analyses

The trunk blood was collected in tubes containing 10 µg of EDTA. The blood was kept on ice before being centrifuged at 3600 rpm for 8 minutes, and 50 uL of plasma was then collected for determination of corticosterone levels. Plasma was immediately frozen at −80°C until analyzed. Corticosterone levels were measured by a commercial radioimmunoassay (RIA) kit (ICN Biomedicals, CA, USA). Inter-assay variability was avoided by assaying all samples (in duplicate) within a single run.

### 2.6 Monoamine Detection

HPLC was performed on collected brain punches to determine levels of central monoamines and metabolites. Brain tissue was sonicated in the homogenizing buffer that they were stored in. Tissue was then centrifuged (15 000 *g* for 20 min), and the supernatants were passed through a radial compression column (5 m, C18 reverse phase, 8 mm×10 cm) connected to a three-cell coulometric electrochemical detector (ESA model 5100, A). Each litre of the mobile phase used for the separation comprised 0.1 g disodium EDTA, 1.3 g heptane sulphonic acid, 35 mL acetonitrile and 6.5 mL triethylamine. The mobile phase was subsequently filtered (0.22-mm filter paper) and degassed, following which phosphoric acid was used to adjust the pH to 2.5. Determination of the area and height of the peaks was carried out with the aid of a Hewlett-Packard integrator. The protein concentration of each sample was determined using bicinchoninic acid with a protein analysis kit (Pierce Scientific, Brockville, Ontario) and a spectrophotometer (Brinkman, PC800 colorimeter).

### 2.7 Western Blot Detection of Hippocampal p-GR

It was of interest to assess whether the JNK antagonist or the p38 antagonist affected hippocampal levels of phosphorylated glucocorticoid receptor (GR; phosphorylated at Ser 234 in mouse). Indeed, hippocampal GR receptors have an important role in the regulation of HPA responses and, as already mentioned, JNK is believed to affect GR phosphorylation status. To this, end Western blot analyses were conducted on whole tissue punches using antibodies directed at the Ser 234 phosphorylation site in mice.

All chemicals were obtained from Sigma-Aldrich (St. Louis, MO) unless otherwise stated. Samples were diluted with lysis buffer containing a protease inhibitor yielding whole cell lysate concentrations of 10 ug of protein in 10 ul and 10 ul loading buffer (5% glycerol, 5% β-mercaptoethanol, 3% SDS and 0.05% bromophenol blue). The 20 ul sample was heated in boiling water for 5 minutes to denature the proteins. Sodium dodecyl sulphate-polyacrylamide gel electrophoresis (SDS-PAGE), the separating buffer (370 mM Tris-base (pH 8.8), 3.5 mM SDS), and the stacking buffer (124 mM Tris-base (pH 6.8), 3.5 mM SDS), were placed in running buffer (25 mM Tris-base, 190 mM glycine, 3.5 mM SDS), and samples along with Precision Plus Protein Standards Dual Color (Bio-Rad, Hercules, CA), were loaded into the Acrylamide gel (8.5%) for molecular weight determination at 120 V.

After electrophoresis, proteins were transferred onto a PVDF (Bio-Rad) overnight at 4°C and 180 mA, in transfer buffer (25 mM Tris-base, 192 mM Glycine, 20% methanol). Membranes were blocked for 1 hour in a solution of non-fat dry milk (5% w/v) dissolved in TBS-T buffer (10 mM Tris-base (pH 8.0), 150 mM sodium chloride, 0.5% Tween-20). Membranes were then incubated with a monoclonal mouse anti-pGR primary antibody (1∶1000; Abcam, Cambridge, MA) diluted in 3% BSA in TBS-T for 1 hour at room temperature. After primary incubation membranes were washed at room temperature in TBS-T three times for a total of 30 minutes. Membranes were then incubated in HRP (horseradish peroxidase) conjugated anti-mouse secondary antibody (1∶5000; Santa Cruz Biotechnology, Santa Cruz, CA) diluted in 5% milk in TBS-T at room temperature for one hour. After three final washes in TBS-T for a total of 30 minutes, pGR was visualized with a chemiluminescent substrate (Perkin Elmer, Waltham, MA; 5 minutes) and exposed on a Kodak film. Band density was quantified using Image J software (National Institutes of Health, Bethesda, MD) and normalized against β-actin.

### 2.8 Statistics

Data were analyzed by 2 (injection: JNK inhibitor/p38 inhibitor or vehicle) by 2 (stress: stress or no stress) analyses of variance. For ANOVAs exhibiting a significant interaction, post hoc analyses using Fisher’s protected least significant difference (Fisher’s PLSD) were conducted to determine the exact nature of the relationship. Data were evaluated using a StatView (version 6.0) statistical software package available from the SAS Institute, Inc.

## Results

### 3.1 Plasma Corticosterone Levels

**Figure 1:**
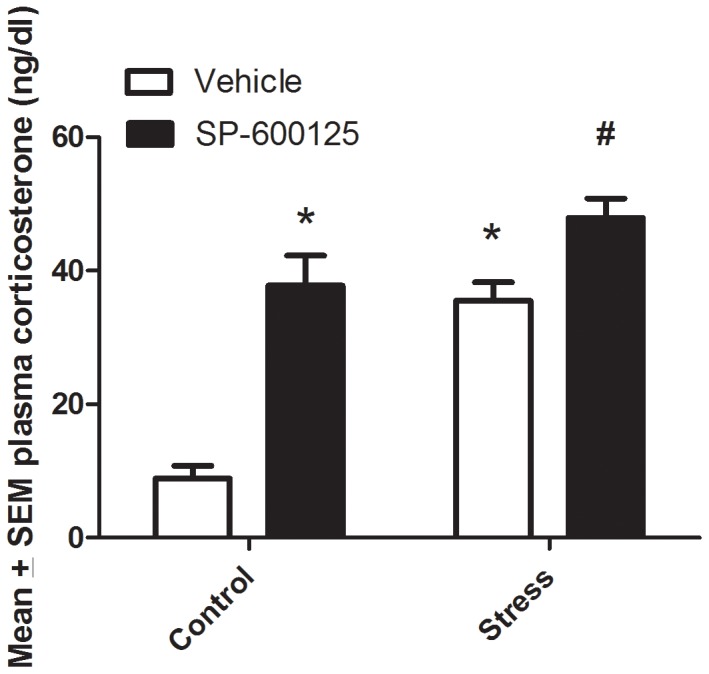
The effect of the JNK antagonist, SP600125, on corticosterone levels varied as a function of stressor treatment. The JNK antagonist and stressor individually increased corticosterone levels. However, the corticosterone rise was most pronounced in mice that received both the JNK antagonist plus the stressor. *p<0.05 relative vehicle control group; ρ *p*<0.05 relative to vehicle stress group. Error bars represent standard error of the mean (SEM).

The effects of the JNK antagonist on corticosterone levels of mice subjected to an acute stressor were assessed. A significant JNK Inhibitor × Stressor interaction was apparent (*F*_1,25_ = 6.024, *p*<0.05). As shown in Fig. 1, post hoc analyses revealed that both the acute stressor and JNK antagonist individually increased corticosterone levels. Moreover, the corticosterone elevation was further enhanced in mice that received both treatments, such that hormonal levels significantly exceed that of the individually treated animals (p<0.05).

**Figure 2:**
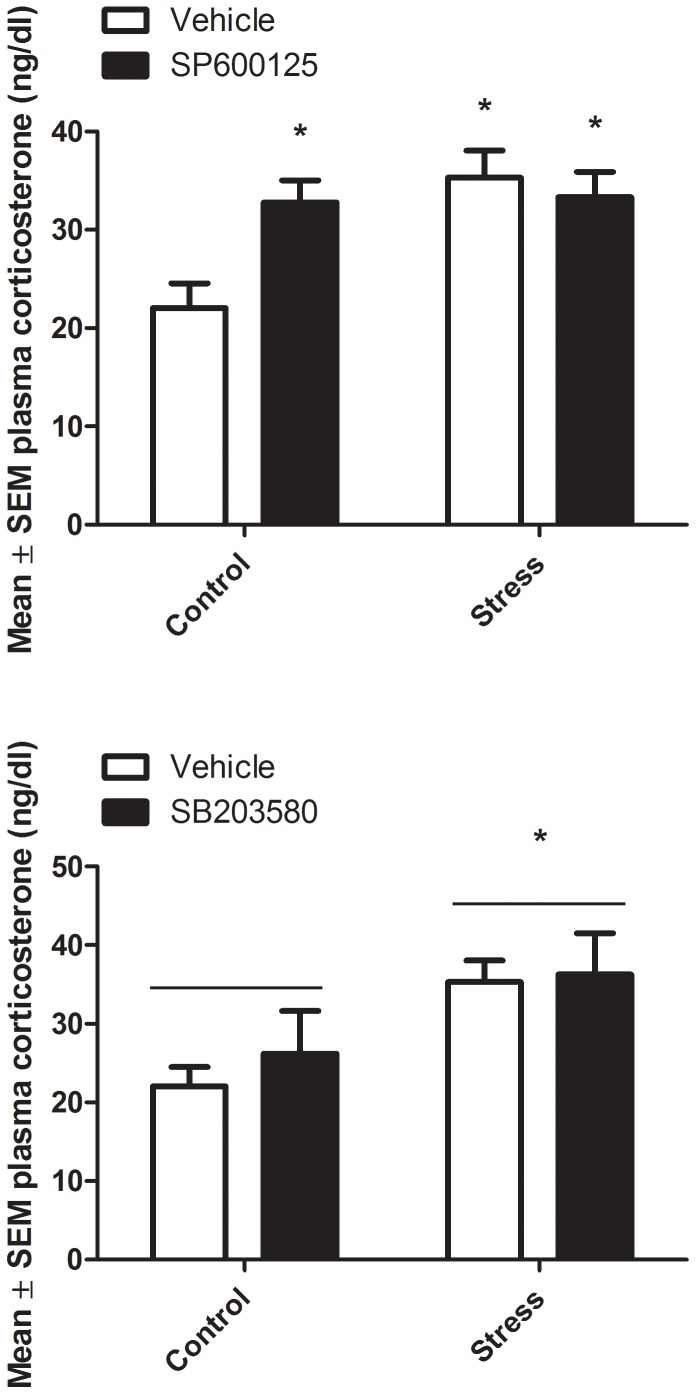
The JNK antagonist, SP600125, and stressor exposure increased corticosterone levels compared to vehicle-treated controls. In contrast, the p38 inhibitor, SB203580, did not affect corticosterone levels in any group. *p<0.05 relative to vehicle control group. Error bars represent standard error of the mean (SEM).

In a separate cohort of animals, the effects of JNK and p38 inhibition on corticosterone levels were examined in mice exposed to an acute stressor. The JNK inhibitor and p38 inhibitor animals were analyzed separately. For the JNK inhibitor, a significant JNK inhibitor x stressor interaction was once again observed (F_1,28_ = 6.37; Fig. 2). Further post hoc analysis revealed that both the JNK inhibitor and the stressor significantly increased corticosterone levels (p<0.05), but there was no further effect of stress on the corticosterone levels of the JNK inhibitor-treated animals. For the p38 inhibitor, there was a significant main effect of stress on corticosterone levels, (F_1, 28_ = 7.63; Fig. 2), wherein the stressor increased levels of corticosterone in both vehicle and p38 inhibitor-treated animals (p<0.05). However, the p38 antagonist had no significant effect on corticosterone levels.

### 3.2 Open Field

**Figure 3:**
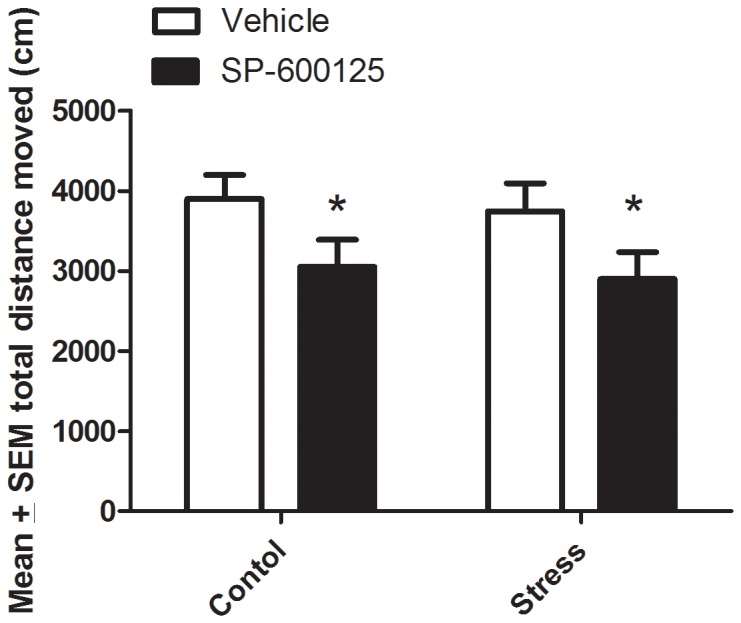
Mice that received the JNK antagonist, SP600125, displayed a significant reduction of total distance moved in the open field compared to vehicle treated controls. *p<0.05. Error bars represent standard error of the mean (SEM).

Although no significant interaction was apparent, a main effect for the JNK antagonist was evident for total distance moved in the open field, (*F*_1,27_ = 5.42; Fig. 3). Specifically, the inhibitor significantly reduced total distance moved compared to vehicle-treated controls in both the stressed and non-stressed mice (p<0.05). However, the JNK antagonist did not significantly influence the number of entries into the inner square compared to vehicle-treated controls in both stressed and non-stressed mice;(p = 0.07, data not shown).

### 3.3 Plus Maze

**Figure 4:**
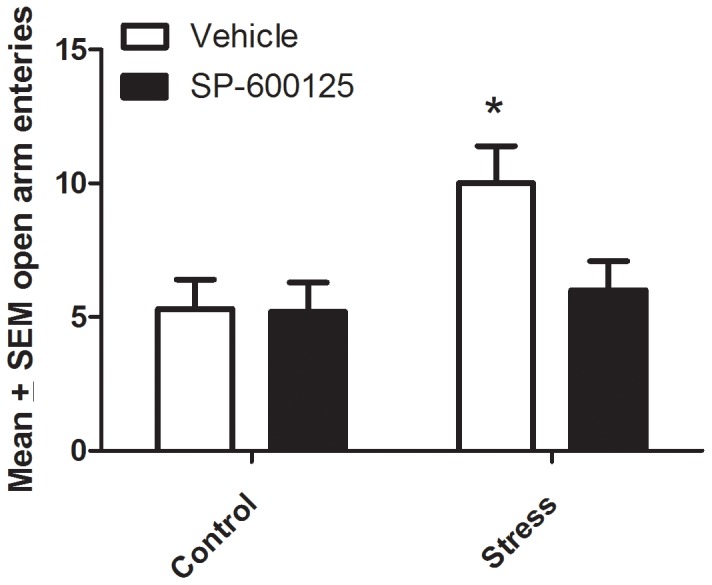
Vehicle treated mice that were subjected to an acute stressor displayed increased entries into the open arms of the plus maze, relative to the non-stressed or JNK antagonist (SP600125)/stress groups, *p<0.05. Error bars represent standard error of the mean (SEM).

Again no signification interaction was apparent for the JNK antagonist and acute stressor. However, there was a main effect for the stressor treatment with regard to the number of entries into the open arms of the elevated plus maze (*F*_1,26_ = 5.13; Fig. 4). The follow up comparisons indicated that there was a significant increase in number of open arm entries evident in the acutely stressed mice (*p*<0.05) and although again no significant interaction was apparent, this effect was restricted to the vehicle treated animals. Indeed, it is clear from Fig. 4 that SP600125 treatment appeared to reverse the stressor induced elevation in open arm entries. In contrast, the stressor and JNK inhibitor did not significantly affect the number of entries into the closed arm of the elevated plus maze.

### 3.4 Central Monoamine Levels

**Figure 5:**
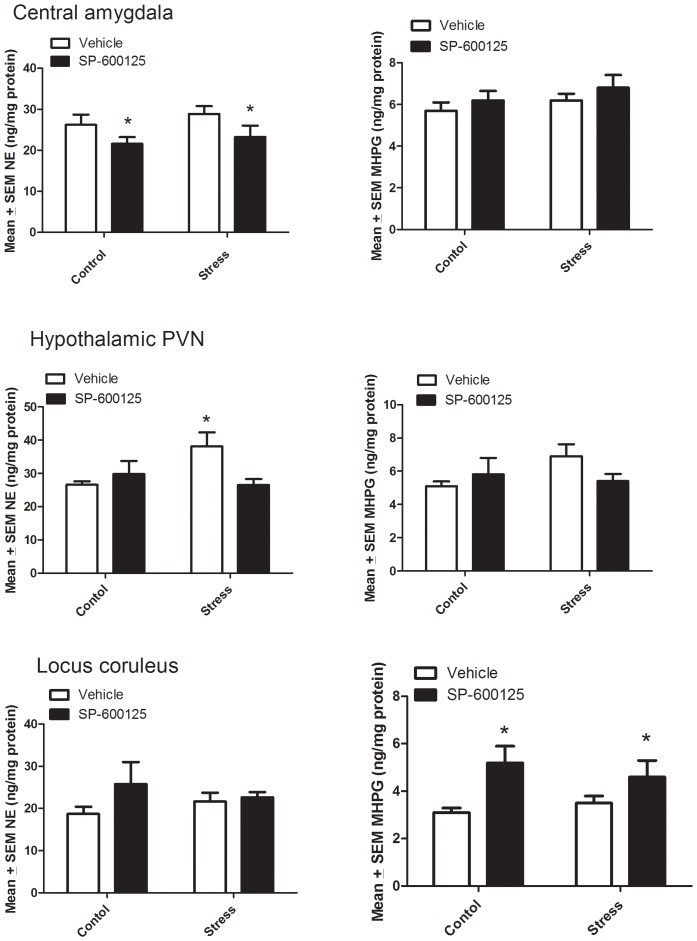
The JNK antagonist, SP600125, significantly reduced norepinephrine (NE) levels in the central amygdala of both non-stressed and stressed animals compared to vehicle-injected controls. The stressor induced elevation of hypothalamic NE levels was prevented by SP600125 treatment. The JNK antagonist significantly increased levels of the NE metabolite, MHPG, in locus coruleus of both non-stressed and stressed mice. *p<0.05 relative to vehicle treated mice.

Levels of norepinephrine (NE) and its metabolite, MHPG, were assessed in the central amygdala, PVN region of the hypothalamus, locus coruleus, as well as the hippocampus and prefrontal cortex (Fig. 5). There were no significant monoamine variations within the prefrontal cortex or hippocampus as a function of the stressor or JNK inhibitor treatments; hence, these data are not shown. Treatment with the JNK antagonist however did provoke significant differences in NE levels within the central amygdala (*F*_1,25_ = 5.00; *p*<0.05; Fig. 5). Specifically, the antagonist reduced NE levels, relative to vehicle treated controls (p<0.05), and this effect was observed in both the stressed and non stressed animals. However, there were no significant differences with regards to MHPG accumulation in the amygdala (Fig. 5).

Within the PVN, the effect of the JNK antagonist on NE levels varied as a function of stress treatment (*F*_1,24_ = 5.41; *p*<0.05; Fig. 5). Indeed, post hoc comparisons revealed that the stressor treatment increased PVN NE levels compared to non-stressed vehicle controls (*p*<0.05); and that this effect was attenuated by the JNK antagonist (*p*<0.05). Again, there were no significant differences in MHPG levels in the PVN following the various treatments (Fig. 5). However, the JNK antagonist did significantly increase MHPG levels within the locus coruleus of both stressed and non-stressed mice (F 1,24 = p<0.05), in the absence of any significant variations of NE levels.

### 3.5 Western Blot

There was no significant difference between mice treated with vehicle and those treated with the JNK antagonist, SP600125, or the p38 antagonist, SP203580, in either of the control and stress conditions, on hippocampal levels of phosphorylated GR (p>0.05; Data not shown).

## Discussion

Stressor related disorders are associated with a wide array of biological changes, including monoamine alterations, deficiencies in neurogenesis and trophic factor signaling, as well as HPA activation [5], [2], [19], [20]. In particular, the enhanced glucocorticoid signaling induced by stressor exposure has profound CNS consequences and might even underlie neuronal damage (e.g. CA1 hippocampal neuron) following chronic exposure [21], [22]. Hence, a better understanding of the molecular pathways contributing to HPA alterations and other negative effects of stress upon CNS functioning is essential for developing novel means of treating stressor-related disorders. In the present paper, we report data suggesting a tonic inhibitory role for the MAP kinase, JNK, in regulating central monoamine utilization, corticosterone release and behavioral activity. At the same time, however, the role of endogenous JNK appears to change in the context of acute stressors, wherein JNK inhibition attenuates certain features of the challenge. Importantly, inhibition of the alternate MAP kinase, p38, had no significant impact upon corticosterone alone or in the context of the stressor, suggesting a selective role for JNK in this regard.

JNKs are increasingly being recognized as playing a role in pathological processes associated with neurological conditions [23], [24], [25], [26], [27]. Yet, JNK activity was also reported to play a positive role in neuroplasticity and recovery from neural injury [28], [29], [30], indicating multi-functional roles for the kinase. Most importantly, it is well established that JNKs are involved in the response to direct cellular stressors such as UV radiation and osmotic shock [31], [32]. Indeed, following stressor exposure JNK can activate numerous stress-regulatory genes by phosphorylating a range of transcription factors, including c-Jun, c-Fos, ATF-2, p53 and c-Myc [33]. Others have reported that JNK activity can also promote phosphorylation of the GR [17], [34], suggesting potential importance in the regulation of neuroendocrine responses to psychological stressors. In this regard, recent studies reported that prenatal stress, as well as chronic (21 days) unpredictable stressor exposure during adulthood reduced the expression of the phosphorylated form of JNK (but not the alternate MAP kinase, ERK) within the hippocampus [35], [36]. Correspondingly, the prenatal stressor (pregnant dams exposed to three sessions of restraint + exposure to a bright light for 45 min) elevated hippocampal and frontal cortex levels of the phosphatase, PP2A, which normally functions to de-phosphorylate (or essentially de-activate) JNK [35].

We currently report that administration of the JNK antagonist, SP600125, greatly increased circulating corticosterone levels. The fact that the JNK antagonist elevated corticosterone levels in both stressed and non-stressed mice suggests that JNK may be regulating basal HPA tone. In this regard, JNKs are known to be involved in the regulation and activity of GRs in the hippocampus, a region intimately involved with HPA axis regulation through negative feedback mechanisms [12]. Moreover, in addition to JNK inhibiting GR expression, the converse also appears to hold true, wherein JNK activity is down-regulated by glucocorticoid binding to GR [34]. Thus, it is possible that bi-directional JNK-GR interactions could normally play a role in modulating basal HPA tone and possibly even reactivity to stressors.

Given that JNK has been reported to phosphorylate GR [17], one would expect JNK inhibition to result in decreased levels of phosphorylated GR. However, we presently did not find any differences in levels of phosphorylated GR within the hippocampus at 45 minutes post JNK inhibition. It is possible that such changes might have been present at other times. Indeed, a previous report indicated that the JNK antagonist provoked c-Jun phosphorylation after 15 min and this effect was absent by 1 hour [37]. Alternatively, another possibility is that we missed subtle changes in phosphorylation that might have been apparent in separate nuclear or cytoplasmic fractions. Indeed, when phosphorylated by JNK, GR is inhibited and remains in the cytoplasm, whereas, when activated, GR translocates to the nucleus where it acts as a transcription factor [12]. We currently used whole hippocampal tissue punches (thereby combining all cellular fractions together), which might have reduced the signal to noise ratio; indeed, increased variability of the p-GR data was certainly an issue in the present data.

Although a peripheral site of action cannot be presently ruled out, it is likely that the JNK inhibitor acted centrally since previous studies indicate that the drug had inhibitory effects in the brain within one hour following intraperitoneal injection [38], [39]. Moreover, JNK3 could be the target underlying the present findings since this is the isoform that is enriched within the brain [14]. Although SP600125 inhibits all three JNK isoforms [40], the fact that no significant difference was found in circulating corticosterone levels in JNK1-deficient mice [41], is consistent with a central JNK3 mechanism controlling HPA responsivity. Yet, one should not discount the possibility that JNK could modulate corticosterone release by acting directly on the adrenal glands since one previous finding did indicate that adrenal JNK expression was induced in response to ACTH binding [42].

The behavioral findings generally support the notion that JNK inhibition can influence exploration within the context of a stressful environment. Indeed, mice systemically injected with the JNK antagonist displayed decreased exploration in an open-field arena. Our open field findings could reflect a number of processes being affected by JNK inhibition, including those related to anxiety, locomotion or general malaise. The fact that the JNK antagonist did not significantly reduce the number of entries into the inner zone of the arena argues against processes linked to anxiety being affected. In contrast, the significant reduction in total distance travelled (and velocity; data not shown) raises the possibility that JNK inhibition was somehow impacting locomotor capability or promoting malaise. Whatever the case, it is unlikely due to any direct neuronal damage given the short timeline of the study and since it has been reported that systemic SP600125 administration actually increased recovery of motor function after spinal cord injury [43], suggesting that the inhibitor does not cause deficits in motor performance. Alternatively, reduced exploration might have been driven by some degree of malaise; however, gross qualitative observation of the animals did not reveal any obvious signs of sickness such as hunched posture, piloerection or ptosis. This is not to say that some “sub-clinical” sickness wasn’t apparent; indeed, we previously found that the corticoid changes elicited by immunological stressors (e.g. LPS) were accompanied by malaise [2]. The previous finding that SP600125 inhibited LPS induced prostaglandin production [44], coupled with the evidence that hypothalamic and peripheral prostaglandins are important for sickness behaviors [45], raises the possibility that such mechanisms were involved in the present behavioral findings. Further adding to the complexity of the issue are findings indicating that JNK antagonist infusion into the CA1 region of the hippocampus did not affect open field behavior [37]; hence alternate brain regions must be of importance, including the possibility of direct actions at the hypothalamus or other limbic regions.

In contrast to open field changes, the JNK antagonist alone did not significantly affect elevated plus maze performance, but it did reverse the stressor induced increased entry into the open arms. This finding seems to be at odds with the open field data, given that it supports a potential anti-anxiety-like effect of JNK inhibition in this particular behavioral test. However, increased entries into open arms in the elevated plus maze has also been reported by many to be a measure of impulsivity [46], [47]. Hence, the increased entries into the open arms may have been a reflection of impulsivity in the group that was exposed to an acute stressor, and JNK inhibition may have interfered with processes important in this regard. At this juncture, we are uncertain as to the exact mechanisms or processes that underlie or contribute to the complex, task-dependent behavioral consequences of JNK inhibition.

In addition to affecting HPA output, the JNK antagonist altered noradrenergic activity in stressor-sensitive brain regions, including the central amygdala, PVN and locus coruleus. The fact that the JNK inhibitor, SP600125, influenced NE activity within the amygdala and locus coruleus independent of any stressor effects, suggests that (like its HPA actions) endogenous JNK is potentially having a tonic regulatory influence over neurochemical activity in these brain regions. It is particularly interesting to note that (in contrast of the hippocampus) the amygdala has long been thought to have a primarily excitatory role upon hypothalamic CRH neurons, thereby facilitating corticosteroid release [48, [49]. Similarly, locus coruleus NE projections are known to impart excitatory effects upon limbic brain regions resulting in enhanced vigilance [50], [51]. Hence, endogenous JNK could normally be acting to restrain such responses.

It is curious to note that the pattern of stressor + SP600125 induced hypothalamic changes paralleled the elevated plus maze changes but was in opposition to the corticoid effects. This divergent pattern of effects appeared to largely stem from the fact that the stressor alone appeared to preferentially affect hypothalamic NE and elevated plus maze behavior. Given that mice treated with the JNK antagonist did not differ from control on these measures suggests that inhibiting endogenous JNK can overcome stressor effects. Yet, in the absence of the stressor, the JNK antagonist itself clearly induced stressor-like effects (at least, with regards to corticosterone and open field exploration). These data indicate that hypothalamic amine responses associated with JNK inhibition were clearly distinguishable from those of the amygdala and locus coruleus; possibly, reflecting a divergent role for JNK in affecting neuroendocrine vs anxiety/vigilance-relevant processes.

### Conclusions

To the best of our knowledge, this is the first report of behavioral, hormonal and neurochemical changes in response to systemic JNK antagonism alone and in the context of a psychologically relevant stressor. Although in stressful situations JNK might contribute to certain behavioral features and monoamine changes, its most robust effects appear to be evident in the absence of a stressor. Our results are consistent with a normal role for endogenous JNK in keeping HPA functioning in check under basal conditions and in the modulation of behavioral and monoamine activity. These data certainly speak to the need for further assessment of the potential importance of JNK and other MAP kinases in the long-term consequences of stressors. It is also important to consider the possibility that selective JNK or MAP kinase targeting drugs could offer new therapeutic agents for treating stressor disorders. Indeed, repeated stressors could sensitize HPA and monoamine activity resulting in psychiatric illness and we speculate that such increased sensitivity would be reflected in an exaggerated basal state that could stem from disruptions in endogenous JNK functioning.
